# Multidimensional synthetic chiral-tube lattices via nonlinear frequency conversion

**DOI:** 10.1038/s41377-020-0299-7

**Published:** 2020-07-20

**Authors:** Kai Wang, Bryn A. Bell, Alexander S. Solntsev, Dragomir N. Neshev, Benjamin J. Eggleton, Andrey A. Sukhorukov

**Affiliations:** 1 Nonlinear Physics Centre, Research School of Physics, The Australian National University, Canberra, ACT 2601 Australia; 2 Institute of Photonics and Optical Science (IPOS), School of Physics, University of Sydney, Sydney, NSW 2006 Australia; 3 Department of Physics, QOLS, Imperial College London, London, SW7 2AZ UK; 4 School of Mathematical and Physical Sciences, University of Technology Sydney, Ultimo, NSW 2007 Australia; 5 Present Address: Ginzton Laboratory, Stanford University, Stanford, CA 94305 USA

**Keywords:** Nonlinear optics, Photonic devices

## Abstract

Geometrical dimensionality plays a fundamentally important role in the topological effects arising in discrete lattices. Although direct experiments are limited by three spatial dimensions, the research topic of synthetic dimensions implemented by the frequency degree of freedom in photonics is rapidly advancing. The manipulation of light in these artificial lattices is typically realized through electro-optic modulation; yet, their operating bandwidth imposes practical constraints on the range of interactions between different frequency components. Here we propose and experimentally realize all-optical synthetic dimensions involving specially tailored simultaneous short- and long-range interactions between discrete spectral lines mediated by frequency conversion in a nonlinear waveguide. We realize triangular chiral-tube lattices in three-dimensional space and explore their four-dimensional generalization. We implement a synthetic gauge field with nonzero magnetic flux and observe the associated multidimensional dynamics of frequency combs, all within one physical spatial port. We anticipate that our method will provide a new means for the fundamental study of high-dimensional physics and act as an important step towards using topological effects in optical devices operating in the time and frequency domains.

## Photonics: Lighting up synthetic dimensions

Studies and applications in physics requiring consideration of more than three dimensions will be assisted by a method for creating “all-optical synthetic dimensions,” allowing deeper exploration of topological effects. The frequencies of interacting light, e.g., can act as the lattice sites in multiple synthetic dimensions in addition to the three dimensions of space. A research team led by Andrey Sukhorukov at The Australian National University developed a theoretical framework for multidimensional lattices wrapped in a tube using light travelling in a single nonlinear waveguide. With collaborators from University of Sydney led by Benjamin Eggleton, they experimentally demonstrated the synthetic dimensions in a nonlinear fibre by implementing light interactions with precisely tailored optical pumps. In addition to assisting research in fundamental physics, the method could have practical applications in quantum communication and information processing.

## Introduction

Discrete photonic lattices constitute a versatile platform for topological photonics^1^, with various implementations employing arrays of evanescently coupled waveguides^2^, metamaterials^3^, and coupled resonators^4^. In these systems, the most commonly considered topological features originate from the dispersion associated with the wave-vector space, where accordingly, the geometrical dimensionality fundamentally limits the degrees of freedom that can contribute to the topological invariant. As such, the possibility of accessing higher geometrical dimensions is a key factor enabling a drastic boost in the manifestations of topological effects. This motivates the rapidly developing field of synthetic dimensions^5,6^, where many schemes for artificially creating extra dimensions have been proposed^7–11^ and experimentally demonstrated^12–16^. In general, higher dimensionality is equivalent to increased connectivity; thus, a multidimensional lattice can be synthesized by lower- or even one-dimensional (1D) lattices with long-range couplings extending beyond the nearest neighbours^17–19^. Importantly, the higher-dimensional formalism can reveal extra nontrivial geometrical and topological properties incorporated into the original 1D wave-vector space. Therefore, an essential yet challenging task to facilitate topological photonics in synthetic dimensions^15^ is the development of artificial lattices exhibiting exotic topological behaviour enabled by effectively larger dimensionality, such as higher-order topological modes^20^.

In topological photonics, beyond the consideration of dimensionality, there are other essential ingredients contributing to the versatile topological phenomena. Of key importance is the boundary condition that has been extensively studied in the well-known bulk-boundary correspondence^9,10,15^. Although these are usually considered for lattices with edges, the periodic boundary conditions in extended lattices are also physically relevant. For instance, in carbon nanotubes, the specific way that the honeycomb lattice gets wrapped into the tube can dramatically impact the material properties; yet, the associated topological characteristics remain largely unexplored. Beyond the most familiar types of zigzag and armchair carbon nanotubes, the most general situation arises in between these two cases, corresponding to a chiral periodic boundary condition, where chiral-tube lattices are formed^21^. Notably, synthetic photonic systems provide diverse and flexible platforms on which these lattices can be artificially arranged, going beyond the natural material arrangements. In particular, it is of fundamental interest to explore various lattice types in addition to honeycombs and analyse the possibility of realizing multidimensional analogues of chiral-tube structures.

Another important ingredient of topological photonics lies in the gauge potential and the associated gauge field due to their fundamental role in describing the movement of charged particles. The realization of artificial gauge fields in photonics has underpinned novel manifestations of light emulating charged-particle dynamics, facilitating the multifaceted aspects of topological photonics such as the breaking of time-reversal symmetry^4,9,15,22,23^, non-reciprocal light guiding^24,25^ and topological lasing^26,27^. In a tight-binding photonic lattice, an artificial gauge field generally corresponds to different phases acquired by light when it couples from site A to site B compared with the opposite path from B to A. These gauge fields can give rise to a flux associated with encircling a geometrical area in 2D or higher-dimensional generalizations, which is essential to many topological models, such as the Landau gauge^9,28^ with globally nonzero flux and the Haldane model^19,29^ with locally nonzero flux.

The temporal and spectral behaviours of light play an important role in many research fields and applications, from telecommunications to the spectroscopy of materials. Importantly, many shortcomings restricting the performance of photonic systems arise in the temporal domain, such as group-velocity dispersion. It is thereby important to achieve topological effects for robust operation in temporal or spectral systems in a regime compatible with common applications, such as frequency combs generated from complementary metal-oxide-semiconductor (CMOS) compatible integrated resonators with a free spectral range (FSR) in the GHz regime^30^. To date, the control of spectral couplings has been primarily studied^9,10,19^ and realized^28,31–33^ based on electro-optic modulation (EOM). However, this approach fundamentally limits the frequency separation between coupled modes by the EOM bandwidth, which commonly restricts the induced coupling to the nearest spectral lines in synthetic frequency lattices. In contrast, all-optical approaches based on parametric nonlinearity^22,34^ and the photon–phonon interaction^35–37^ appear to be promising solutions to the task of bringing multidimensional topological photonics to devices requiring ultra-fast temporal modulation and accordingly large FSR. Recently, we reported the implementation of synthetic long-range coupling in an all-optical system mediated by parametric nonlinearity within one spatial mode (port) with up to 100 GHz separation between the spectral lines^22^. However, the possibility of realizing multidimensional synthetic lattices in an all-optical platform remains unexplored.

In this work, for the first time to our knowledge, we theoretically establish and experimentally demonstrate that all-optical spectral lattices can synthesize multidimensional chiral lattices in combination with nontrivial gauge fields. The synthetic dimensions are based on simultaneous specially tailored short- and long-range couplings between discrete frequency components, which are mediated by optical nonlinearity and are directly controlled by the spectral shape of the optical pump. With three orders of coupling, we show the construction of triangular chiral-tube lattices in three dimensions and establish their four-dimensional (4D) generalization. We also develop a pump configuration that induces nontrivial artificial gauge fields associated with effective nonzero magnetic flux and experimentally demonstrate their influence on a quantum walk in triangular chiral-tube lattices.

We note that our all-optical implementation achieves a broad operating bandwidth of hundreds of GHz, which exceeds the capabilities of the complex EOM schemes considered previously while offering greater simplicity. The high bandwidth enables direct matching with the FSR of integrated resonators, which can facilitate multiple applications of the synthetic multidimensional lattices presented in our work. Moreover, a capability for the multidimensional and coherent reshaping of discrete frequency lines can enable the unconventional and non-reciprocal manipulation of quantum frequency combs^38^, which may boost the capacity of photonic quantum communications and information processing.

## Results

### Construction of synthetic dimensions in a nonlinear waveguide

**Fig. 1:**
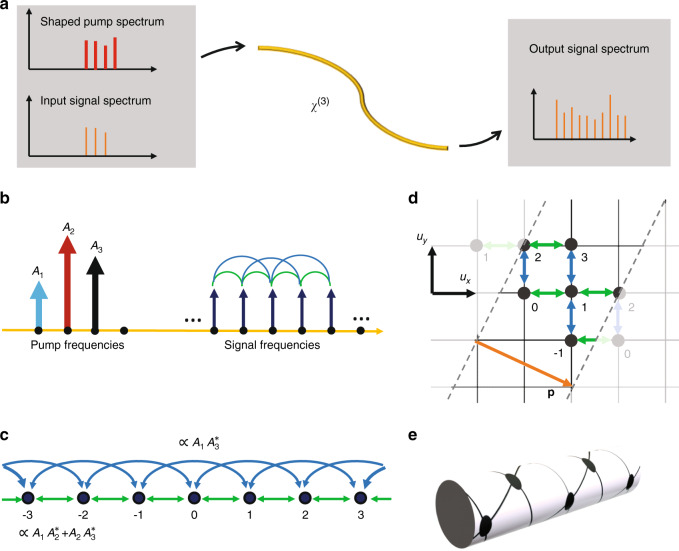
Conceptual sketch of constructing multidimensional synthetic lattices in a nonlinear fibre. **a** A nonlinear waveguide with *χ*^(3)^ nonlinearity, where a shaped pump mediates the conservative interactions between signal frequencies, giving rise to a reshaped signal spectrum at the output. **b** An example of a pump profile that induces cross-talk between one and two unit frequency separations. **c** The corresponding spectral lattice with first-order (*C*_1_) and second-order (*C*_2_) couplings driven by nonlinear interactions mediated by the shaped pump spectrum shown in **b**. **d** Synthetic two-dimensional square lattice constructed using the spectral lattice in **c**. **e** Illustration of the chiral-tube lattice formed by wrapping the lattice in **d** with the chiral periodic boundary condition **p**

We start by introducing a general approach for the implementation of spectral photonic lattices in a nonlinear waveguide. As sketched in Fig. 1a, a spectral lattice is realized by using a shaped pump composed of several equidistant frequencies with spacing Ω. This pump can drive the interactions between the frequency components on the input signal spectrum all inside one fibre or waveguide with *χ*^(3)^ nonlinearity in the regime of the so-called four-wave-mixing Bragg scattering^22^. Under energy conservation and undepleted pump approximation, each pair of pump frequencies separated by *n*Ω drives the coupling between two signal lines with the same frequency difference *n*Ω, as shown in Fig. 1b. These discrete spectral lines form a lattice (Fig. 1c), where each frequency represents one lattice site. Importantly, nonlocal and complex-valued couplings can be implemented by specially tailoring the pump spectrum^22^.

Now, we outline the key concept of exact mapping between higher-dimensional lattices and a 1D spectral lattice with nonlocal couplings induced through nonlinear frequency conversion. In the example shown above in Fig. 1a–c, three pumps equally separated by Ω introduce coupling of the first and second orders (Fig. 1c). Then, the evolution of the signal spectrum along the nonlinear waveguide in the phase-matching regime is governed by the Hamiltonian in terms of the creation ($\hat a_m^\dagger$) and annihilation ($\hat a_m$) operators for the discrete signal frequency components:

1 $${\mathbf{H}} = - \mathop {\sum }\limits_m \,\mathop {\sum }\limits_{\{ n\} } \,C_n\hat a_m^\dagger \hat a_{m + n} - {\mathrm{H}}.{\mathrm{c}}.$$

where a set of positive integers {*n*} indicates the orders of coupling and *C*_*n*_ are the corresponding coupling constants of the *n*-th order. ‘H.c.’ denotes the Hermitian conjugate, and *m* is an integer running through all phase-matched spectral lines. The coupling constants *C*_*n*_ are given by the following expression^22^:

2 $${\it{C}}_{\it{n}} = 2\gamma {\it{P}}\mathop {\sum}\nolimits_{\it{m}} {{\it{A}}_{\it{m}}{\it{A}}_{{\it{m}} - {\it{n}}}^ \ast }$$

where *γ* is the effective nonlinearity and *P* is the average pump power. Here, *A*_*m*_ denotes the complex amplitudes of pump spectral components in the fibre, which are normalized as $\mathop {\sum}\nolimits_m {|A_m|^2 = 1}$. The evolution of the wavefunction governed by the Hamiltonian in Eq. (1) can be expressed as

3 $$\psi (z) = {\mathrm{exp}}(iz{\mathbf{H}}) = {\mathrm{exp}}(izP{\mathbf{H}}^{\prime} )$$

where *z* is the propagation distance along the fibre and we denote by **H′** = **H**/*P*, a normalized Hamiltonian that is independent of the total pump power. We see that the wavefunction dynamics can be observed by varying the average pump power *P* for a fixed fibre length *z* = *L* such that *P* effectively acts as the time variable.

We now consider a nontrivial and representative case of two coupling orders *n* = 1,2 and show how the spectral lattice is mapped to a two-dimensional square lattice. The general idea is based on the mapping of each specific order of coupling to a certain basis vector in higher-dimensional space. For the example shown in Fig. 1d, which is a two-dimensional space of a square lattice, there are two basis vectors, **u**_x_ and **u**_y_. Hence, we can map the coupling order *n* = 1 to **u**_**x**_ and *n* = 2 to **u**_y_. Then, we obtain a Hamiltonian in the two-dimensional space that represents a square synthetic lattice:

4 $${\mathbf{H}}_{{\mathbf{sq}}} = - \mathop {\sum}\nolimits_m {\left[ {C_1\hat a_{{\mathbf{r}}_{\mathbf{m}}}^\dagger \hat a_{{\mathbf{r}}_{\mathbf{m}} + {\mathbf{u}}_{\mathbf{x}}} + C_2\hat a_{{\mathbf{r}}_{\mathbf{m}}}^\dagger \hat a_{{\mathbf{r}}_{\mathbf{m}} + {\mathbf{u}}_{\mathbf{y}}}} \right] - {\mathrm{H}}.{\mathrm{c}}.}$$

where **r**_**m**_ is a vector indicating the spatial coordinate of the *m*-th site in this two-dimensional space. To provide an exact mapping, it is essential to reflect in 2D the algebraic property of the 1D lattice, where a sequence of two first-order couplings produces the same frequency shift 2**Ω** as that of a second-order coupling. This property can be satisfied by imposing a periodic boundary condition for the two-dimensional synthetic space, as shown in Fig. 1d, where the orange arrow represents the wrapping vector **p** = 2**u**_**x**_–**u**_**y**_. Consequently, the two-dimensional equivalent lattice is actually wrapped into a (2,−1) chiral tube connected by the dashed lines in Fig. 1d. This chiral tube is schematically illustrated in Fig. 1e.

### Observation of a quantum walk in synthetic triangular lattices

**Fig. 2:**
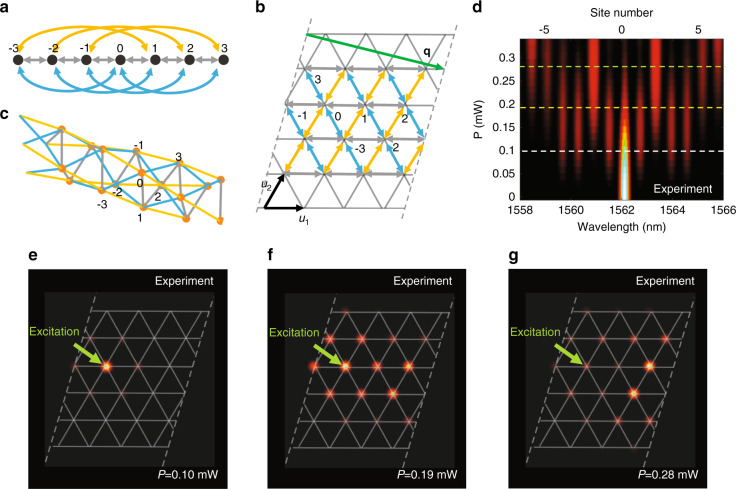
Experimental observation of a quantum walk in a synthetic two-dimensional triangular chiral-tube lattice. **a** A lattice with three coupling orders 1, 3 and 4, where only couplings to the shown sites are plotted with arrows. **b** The corresponding synthetic triangular lattice in two-dimensional space. **c** 3D sketch of the lattice in **b**. **d** Experimental realization of a frequency quantum walk, where *P* is the power of the three-pump spectral components with *A*_1_ = *A*_2_ = *A*_5_. **e**–**g** Mapping of experimental data from **d** to a two-dimensional triangular lattice with *P* *=* 0.10, 0.19, 0.28 mW, as indicated by the labels

We formulate and experimentally demonstrate an original mapping procedure for the realization of a synthetic *triangular* lattice. This presents a nontrivial case with non-orthogonal basis vectors, which has not been considered on any synthetic photonic lattice platform in previous studies. We show that a triangular lattice can be obtained by mapping from a spectral lattice with specially engineered simultaneously short- and long-range couplings. The synthetic frequency space is sketched in Fig. 2a, where the first, third and fourth orders of the coupling are present. In the two-dimensional space of the triangular lattice, as shown in Fig. 2b, the basis vectors are **u**_**1**_ = [1,0]^T^ and ${\mathbf{u}}_{\mathbf{2}} = \left[ {1/2,\sqrt 3 /2} \right]^{\mathrm{T}}$, which are not orthogonal relative to each other. We map the first-order coupling to the vector **u**_1_ and the fourth-order coupling to **u**_2_. Then, we find that the third-order coupling is automatically mapped to **u**_3_ = **u**_2_ − **u**_1_. This arrangement is used to construct the two-dimensional equivalent triangular lattice sketched in Fig. 2b with the Hamiltonian

5 $${\mathbf{H}}_{{\mathbf{tr}}} = - \mathop {\sum}\nolimits_m {\left[ {C_1\hat a_{{\mathbf{r}}_{\mathbf{m}}}^\dagger \hat a_{{\mathbf{r}}_{\mathbf{m}} + {\mathbf{u}}_1} + C_3\hat a_{{\mathbf{r}}_{\mathbf{m}}}^\dagger \hat a_{{\mathbf{r}}_{\mathbf{m}} + {\mathbf{u}}_3} + C_4\hat a_{{\mathbf{r}}_{\mathbf{m}}}^\dagger \hat a_{{\mathbf{r}}_{\mathbf{m}} + {\mathbf{u}}_2}} \right] - {\mathrm{H}}.{\mathrm{c}}.}$$

Similar to the example of the square lattice discussed above (Fig. 1d), there appears a periodic boundary condition. It is defined by the wrapping vector **q** = 4**u**_**1**_ − **u**_**2**_, shown as a green arrow in Fig. 2b. Hence, the triangular lattice is effectively wrapped and connected by the dashed lines in Fig. 2b. To show this more intuitively, we sketch a three-dimensional (3D) visualization of the (4,−1) chirally wrapped tube in Fig. 2c. We determine the unit cell vector of the tube lattice as **l** = −2**u**_**1**_ + 7**u**_**2**_ (not shown in the figure), which is the shortest vector that can connect two sites along the parallel direction of the tube (**l**^T^**q** = 0 due to orthogonality).

We now present an experimental realization of a quantum walk in the multidimensional synthetic lattice space. Quantum walks have been observed in various types of photonic lattices using classical laser sources, where the evolution of coherent light is mathematically analogous to the quantum single-particle dynamics^39^. We tailor the complex amplitudes *A*_*m*_ of the pump spectral lines to induce the desired couplings in the signal frequency lattice according to Eq. (2). Specifically, we employ three pumps with equal amplitudes *A*_1_ = *A*_2_ = *A*_5_ to achieve the frequency lattice, as illustrated in Fig. 2a, with equal first-, third- and fourth-order couplings, i.e., *C*_1_ = *C*_3_ = *C*_4_. We shape the pump with no phase difference between the complex amplitudes at different frequencies and, therefore, all couplings are real-valued. With a single-frequency signal excitation, we observe a quantum walk in this frequency space, as shown in Fig. 2d. We map this experimentally realized synthetic lattice to the triangular lattice as outlined in Fig. 2b, c. The mapped quantum walk is shown in Fig. 2e–g at three representative average pump powers *P* = 0.10, 0.19, 0.28 mW, respectively. As mentioned above, the pump power acts as the time variable in the quantum walk. In these figures, the site of excitation is marked by a green arrow. This represents an experimental observation of quantum walks in higher synthetic dimensions. Our results agree quite well with the corresponding theoretical predictions calculated by the coupled mode equations. An animated image illustrating the dynamics of the experiment (incorporating Fig. 2e–g) and a comparison with theory is provided as Supplementary Fig. S1 in supplementary files.

**Fig. 3:**
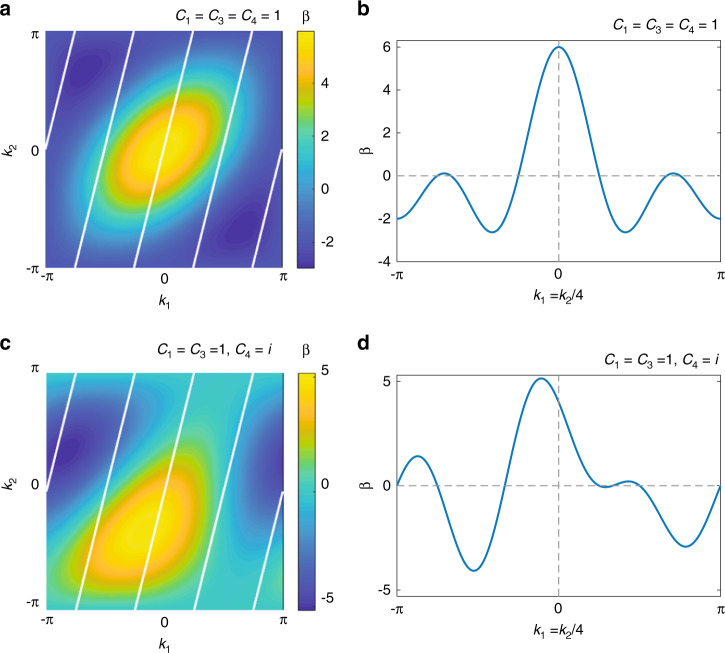
Dispersion of the triangular lattice wrapped in a (4,−1) chiral tube. **a** Colour density plot of the propagation constant *β* vs. wave numbers *k*_1_ and *k*_2_, where the (4,−1) chiral periodic boundary condition allows only *k*_1_,*k*_2_ values on the white lines. Here, we set *C*_1_ = *C*_3_ = *C*_4_ = 1. **b** The corresponding dispersion of **a** plotted in 1D along *k*_1_ = *k*_2_/4. **c**, **d** Plots analogous to **a**, **b** but with a complex coupling *C*_4_ = *i*; the other parameters are the same

To provide insight into the properties of our mapped synthetic chiral-tube lattice, we also perform a theoretical analysis of the wave dispersion. We apply Bloch theorem and calculate the propagation constant as $\beta (k_1,k_2) = 2{\mathrm{Re}}[C_1{\mathrm{exp}}(ik_1) + C_4{\mathrm{exp}}(ik_2) + C_3{\mathrm{exp}}(ik_3)]$, where *k*_1_, *k*_2_ and *k*_3_ are the wave numbers in the reciprocal space of the basis vectors **u**_1_, **u**_2_, and **u**_3_, respectively, and $k_3 \equiv k_2 - k_1$. In Fig. 3a, we plot a representative case for *C*_1_ = *C*_3_ = *C*_4_ = 1. Due to the periodic boundary condition, not all values of *k*_1_,*k*_2_ are allowed. For the (4,−1) chiral tube discussed above, we have 4*k*_1_ − *k*_2_ = 2*Nπ*, where *N* is an integer. These allowed values are denoted as white lines in Fig. 3a. We trace out *k*_1_ = *k*_2_/4 for the range of −*π* to *π* and present the dispersion as a 1D curve in Fig. 3b. For comparison, we also show another case with a different coupling *C*_4_ = *i* in Fig. 3c. We see that the resulting 1D dispersion shown in Fig. 3d becomes asymmetric due to the complex coupling *C*_4_, which breaks the time-reversal symmetry. We show in the following section that this regime is associated with the appearance of a gauge field in the mapped high-dimensional lattices.

### Artificial gauge field with nonzero magnetic flux in chiral-tube lattices

We demonstrate that the complex-valued nature of the coupling constants in the synthetic frequency lattice can enable artificially created gauge fields. In contrast to the 1D case, in higher-dimensional lattices, an important aspect of the gauge potential is associated with the induced magnetic flux that can arise in the presence of nonzero phase accumulation around a closed loop in the lattice.

**Fig. 4:**
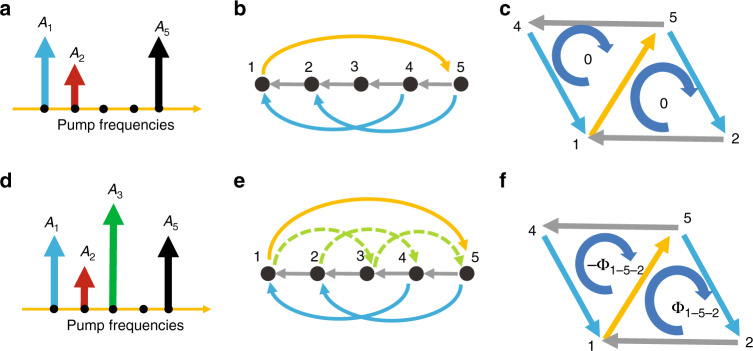
Implementation of the nontrivial gauge field in synthetic triangular lattices. **a** Three-pump configuration used to induce three orders of coupling. **b** The implemented synthetic frequency lattice with the first, third, and fourth orders of coupling. **c** The mapping of **b** to a two-dimensional triangular lattice where the flux in each cell is zero. **d–f** Our specially designed scheme for implementing nonzero flux by adding an extra pump (*A*_3_) and tailoring the pump phases

To illustrate the capacity of our scheme to synthesize a nontrivial gauge field, we first revisit the mapping of a spectral lattice with three orders of coupling, as sketched in Fig. 2a. To induce these couplings, the minimum number of pumps is three, with complex amplitudes *A*_1_, *A*_2_, and *A*_5_, as illustrated in Fig. 4a, with the corresponding couplings $C_1 \propto A_2A_1^ \ast$, $C_3 \propto A_5A_2^ \ast$ and $C_4 \propto A_5A_1^ \ast$. In Fig. 4b, we show a section of a spectral lattice implemented by the pump configuration in Fig. 4a, visualizing five sites (1–5) as an illustration. We use a one-way arrow to show each order of coupling, where the coupling to the other direction simply takes the complex-conjugate value due to Hermiticity. We determine the phases of each order of the coupling along the direction of the arrows in Fig. 4b as *ϕ*_1_ = arg(*A*_2_) − arg(*A*_1_) (grey arrow), *ϕ*_3_ = arg(*A*_5_) − arg(*A*_2_) (blue arrow), and *ϕ*_−4_ = arg(*A*_1_) − arg(*A*_5_) (orange arrow). This lattice is mapped to the triangular lattice using the approach described above, which is shown in Fig. 4c for the first five sites. We find that the clockwise flux vanishes in each of the triangular cells:

6 $$\begin{array}{*{20}{c}} {{\mathrm{\Phi }}_{1 - 5 - 2}}={\phi _{ - 4} + \phi _3 + \phi _1 = 0} \\ {{\mathrm{\Phi }}_{1 - 4 - 5}}={- \phi _{ - 4} - \phi _3 - \phi _1 = 0} \end{array}$$

This shows that with the minimum necessary number of three pumps, the number of free parameters is not sufficient to implement nonzero flux in any of the triangular cells of this two-dimensional lattice.

We reveal that a nonzero flux can be induced by adding an extra pump, indicated with the green arrow in Fig. 4d with amplitude *A*_3_. The corresponding couplings between the signal frequencies are sketched in Fig. 4e. As we are still aiming for coupling orders 1, 3, and 4, we first need to ensure that the second-order coupling, denoted by the green dashed arrows in Fig. 4e, is cancelled out:

7 $$C_2 \propto A_3A_1^ \ast + A_5A_3^ \ast = 0$$

As a sufficient condition to fulfil Eq. (7), in our experiment, we take $|A_1| = |A_5|$, ${\mathrm{arg}}(A_3A_1^ \ast ) = \pi /4$ and ${\mathrm{arg}}(A_5A_3^ \ast ) = - 3\pi /4$. Then, we calculate the phases of the other orders of coupling and find that *ϕ*_3_ (blue arrow) and *ϕ*_−4_ (orange arrow) remain the same as in the case analysed above with three pumps. This situation occurs, as each of the two orders is induced by the same pair of pumps. Importantly, the first-order coupling acquires a different phase according to the expression

8 $$C_1 \propto A_2A_1^ \ast + A_3A_2^ \ast$$

This relation allows us to implement an arbitrary phase *ϕ*_1_ = arg(*C*_1_) in the experiment, where we fix ${\mathrm{arg}}(A_3A_1^ \ast ) = \pi /4$ but freely choose the amplitudes of all three involved pumps and the phase of *A*_2_. Therefore, the limitation given in Eq. (6) no longer applies and we can engineer any nonzero flux Φ_1−5−2_. It is noteworthy that the following condition still holds:

9 $${\mathrm{\Phi }}_{1 - 4 - 5} \equiv - {\mathrm{\Phi }}_{1 - 5 - 2}$$

which leads to a zero flux if one encircles a pair of neighbouring cells. This case is analogous to that of the Haldane model^29^, where the total flux over all cells is zero, yet locally there appear locations with nonzero flux.

**Fig. 5:**
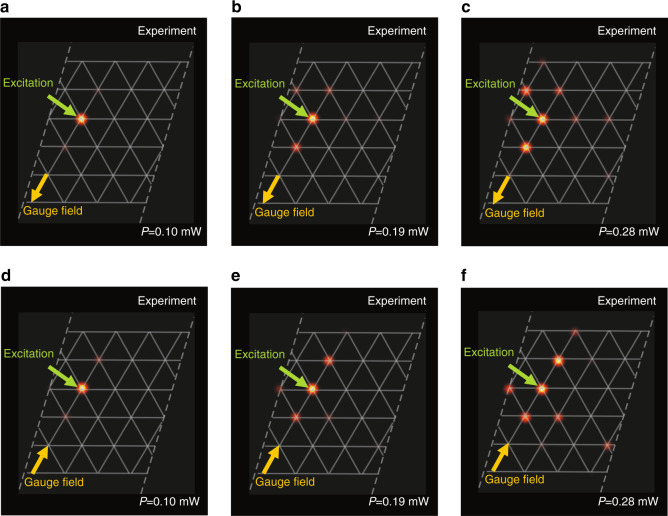
Experimental observation of quantum walks in synthetic dimensions with artificial gauge fields. **a**–**c** Experimental results for an effective complex coupling phase *π*/2 along the direction of the yellow arrow for pump average powers *P* = 0.10, 0.19, 0.28 mW, respectively. **d**–**f** The corresponding cases of **a**–**c** with the opposite direction of the *π*/2 phase

Next, we show a representative set of experimental results that demonstrate these nontrivial gauge potentials. We intentionally make $|C_4|$ slightly larger than $|C_1| = |C_3|$ to more clearly observe the features associated with the artificial gauge potential. Specifically, we choose the pump profiles with four frequencies to obtain the coupling constants $C_1:C_3:C_4 = 3: - 3:5{\mathrm{exp}}(i\alpha )$. This arrangement effectively corresponds to a phase of *π* − *α* along the **u**_2_ basis vector (positive direction) if we use a gauge transformation to make all couplings other than those along **u**_2_ real-valued. In the two sets of experiments presented in Fig. 5, we realize quantum walks with a single-site excitation in the synthetic triangular lattice with *α* = −*π*/2 and *α* = *π*/2. In Fig. 5a–c, we show the case with *α* = *−π*/2, where the yellow arrow indicates the direction along which there is a positive *π*/2 phase in the coupling. For the gradually increasing pump powers, as indicated in Fig. 5a–c, we find that the evolution of the single-site excitation exhibits an asymmetric behaviour along the direction of the effective gauge field. This situation can be clearly seen by a comparison with the case of *α* = *π*/2, which is shown in Fig. 5d–f for the same pump powers as in Fig. 5a–c, respectively. In particular, the patterns formed in the quantum walk, as shown in Fig. 5c, f, look like two arrows pointing in opposite directions. Comparisons of the experimental results with theory for Fig. 5a–c, d–f are shown using two animated images in the supplementary files; see Supplementary Figs. S2 and S3, respectively.

### Higher-dimensional analogues of tube lattices

**Fig. 6:**
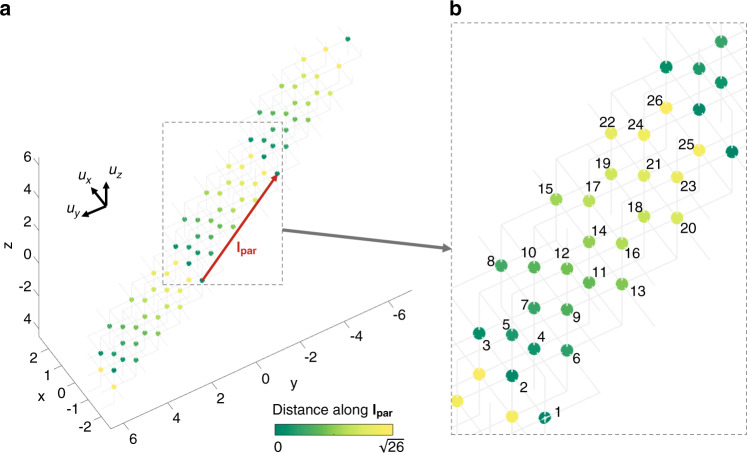
Construction of a four-dimensional analogue of chiral-tube lattices. **a** Sites of a cubic lattice constructed from the coupling orders 1, 3 and 4, which is wrapped in a fourth dimension, forming a chiral lattice. **b** Zoomed-in view of the dashed panel in **a**, with the sites in one unit cell numbered from 1 to 26

We now discuss how 3D cubic lattices can be constructed through mapping and how the periodic boundary conditions wrap the cubic lattice into a 4D analogue of the 3D chiral tubes considered above. We keep using the lattice configuration in Fig. 2a as an example, which involves coupling orders 1, 3 and 4; however, we perform a different mapping procedure. We map the first-order coupling to the basis vector **u**_**x**_, the third-order coupling to **u**_**z**_ and the fourth-order coupling to −**u**_**y**_; see Fig. 6a. By doing so, we effectively realize the Hamiltonian

10 $${\mathbf{H}}_{{\mathbf{cub}}} = - \mathop {\sum}\nolimits_m {\left[ {C_1\hat a_{{\mathbf{r}}_{\mathbf{m}}}^\dagger \hat a_{{\mathbf{r}}_{\mathbf{m}} + {\mathbf{u}}_{\mathbf{x}}} + C_4\hat a_{{\mathbf{r}}_{\mathbf{m}}}^\dagger \hat a_{{\mathbf{r}}_{\mathbf{m}} - {\mathbf{u}}_{\mathbf{y}}} + C_3\hat a_{{\mathbf{r}}_{\mathbf{m}}}^\dagger \hat a_{{\mathbf{r}}_{\mathbf{m}} + {\mathbf{u}}_{\mathbf{z}}}} \right] - {\mathrm{H}}.{\mathrm{c}}.} $$

The mapped cubic lattice is subject to a nontrivial periodic boundary condition. In contrast to the 2D case, where the periodic boundary is described by a wrapping vector, as discussed above, the condition can be expressed here as a wrapping plane **s**, given by the equation *x* − 4*y* + 3*z* = const. Within each wrapping plane, we keep lattice sites with no repetitions, which gives rise to the lattice structure shown in Fig. 6a. We determine the unit cell vector as **l**_**par**_ = **u**_**x**_ − 4**u**_**y**_ + 3**u**_**z**_, which is shown as a red arrow in Fig. 6a. The colour map in Fig. 6a shows the coordinate of each lattice site in a unit cell along **l**_**par**_. We further zoom in the dashed panel in Fig. 6a and show it as Fig. 6b, where we number all 26 sites in one unit cell. It is noteworthy that although this lattice structure is visualized in 3D, there are couplings (connections) enabled by a fourth dimension analogous to the wrapping of a tube. For example, in this 3D layout, site 15 has only two neighbouring sites, i.e., 14 and 19 (see Fig. 6b); however, it actually also interacts with sites 11, 12, 16, 18 and 19 (connections not shown) via a fourth dimension. This is the first example of using a synthetic lattice to realize a 4D generalization of tube lattices. We note that the geometry of this structure is equivalent to an open 3-torus^40^, which is one of the important models used to study the topology of the universe^41^.

## Discussion

To summarize, we have theoretically constructed and experimentally realized nonlinearity-induced synthetic frequency lattices, in which mapping forms multidimensional chiral-tube lattices. The working principle based on nonlinear frequency conversion enables an all-optical realization with large separation between the spectral lines, overcoming the bandwidth limitations of systems employing EOM. We observed quantum walks in artificial two-dimensional triangular lattices wrapped into a 3D tube and implemented gauge fields with nonzero flux. We also showed the construction of a 4D analogue of tube lattices employing the chiral periodical boundary conditions formed in a mapped 3D cubic lattice. We point out that the periodic boundary condition, which is an important ingredient of this work, can also be suppressed if so desired by using a combination of large and incommensurate coupling orders. An interesting open question is how to develop a general mathematical formalism that establishes a mapping between 1D lattices with arbitrary coupling ranges and higher-dimensional lattices with different lattice types. We anticipate that our general conceptual approach may be implemented in a variety of optical setups, including EOM, and can also stimulate new realizations of lattices in the spatial domain^15,16,42^. Furthermore, synthetic lattices can enable new applications for single-shot reconstruction of the amplitude, phase and coherence of signal spectra^43^.

We further note that by employing the process of sum-frequency generation mediated by the second-order nonlinearity, one could implement two sub-lattices that realize a synthetic honeycomb lattice. The on-site potential of each lattice site would be determined by the phase mismatch, which can enable the implementation of edges by dispersion engineering of the nonlinear waveguide, opening a path towards the exploration of topological properties associated with edge-boundary correspondence. Parametric nonlinearity can also be used to realize gain and induce synthetic lattices with non-Hermitian topological properties. Importantly, our approach is, in general, non-reciprocal, as the phase-matching condition is fulfilled in one direction of the nonlinear waveguide determined by the pump^44^; yet, this system is free of the limitations imposed by dynamic reciprocity^45^ associated with nonlinear devices. In addition, as our approach is mediated by optical nonlinearity, it is naturally suitable for the exploration of nonlinear effects in synthetic space, such as multidimensional solitons^46^. Future work may also consider developing schemes to implement other types of synthetic gauge fields in synthetic space, such as those corresponding to a uniform magnetic field that can induce a circular motion of wavepackets in photonics^47,48^. Above all, we anticipate that our work can motivate new fundamental advances and practical experiments in multidimensional topological, nonlinear and quantum photonics.

## Materials and methods

### Experimental setup for a synthetic frequency lattice in a nonlinear fibre

The nonlinear frequency conversion was realized in a highly nonlinear fibre with a length of 750 m, the zero-dispersion wavelength of which was 1551 nm. The coherent light source was a mode-locked laser with an approximately 25 nm bandwidth, which was reshaped by two spectral pulse shapers (Finisar WaveShaper 4000 S), following the approach of ref. ^22^. In the first wave shaper, the laser was split into two channels, with one as a pump channel and the other as a signal channel. The pump channel was then launched into an erbium-doped fibre amplifier (EDFA) for amplification, followed by a variable attenuator to change the average pump power. The signal channel went through a tuneable delay line, which could be adjusted for the signals to match the pump in time. The two channels were then recombined using the second wave shaper, which also shaped the required signal profile (as the input state of the spectral lattice) and removed the noise induced by the EDFA to the pump. The output spectra were observed with an optical spectrum analyser.

## Supplementary information

Supplemetary Animated Figure S1

Supplemetary Animated Figure S2

Supplemetary Animated Figure S3
