# Supplementary figures and images for: Multidimensional synthetic chiral-tube lattices via nonlinear frequency conversion
Source: Light Sci Appl. 2020 Jul 20;9:132. doi: 10.1038/s41377-020-0299-7 (PMC7371864; doi:10.1038/s41377-020-0299-7)

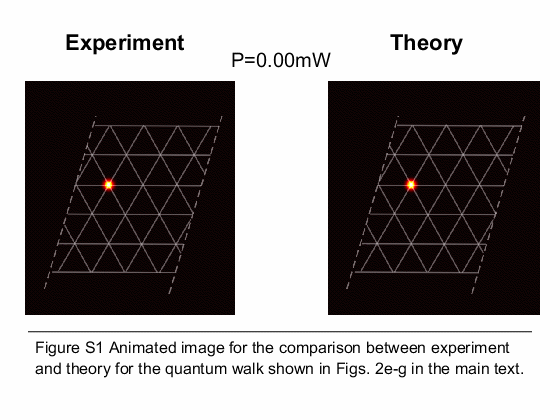

Supplement: Supplementary file 1 — Supplemetary Animated Figure S1 [file 41377_2020_299_MOESM1_ESM.gif]

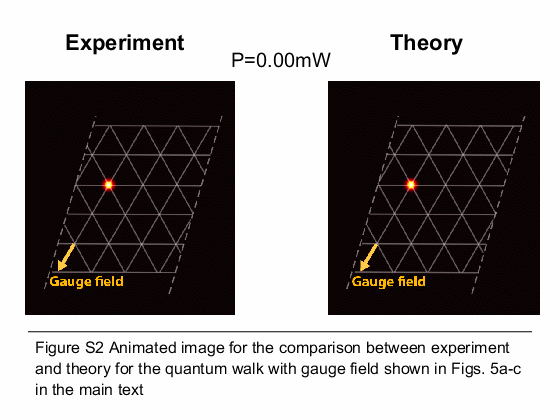

Supplement: Supplementary file 2 — Supplemetary Animated Figure S2 [file 41377_2020_299_MOESM2_ESM.gif]

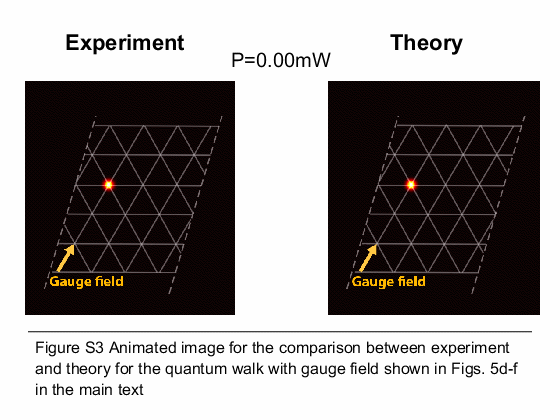

Supplement: Supplementary file 3 — Supplemetary Animated Figure S3 [file 41377_2020_299_MOESM3_ESM.gif]
